# Identification and diagnosis of meniscus tear by magnetic resonance imaging using a deep learning model

**DOI:** 10.1016/j.jot.2022.05.006

**Published:** 2022-06-26

**Authors:** Jie Li, Kun Qian, Jinyong Liu, Zhijun Huang, Yuchen Zhang, Guoqian Zhao, Huifen Wang, Meng Li, Xiaohan Liang, Fang Zhou, Xiuying Yu, Lan Li, Xingsong Wang, Xianfeng Yang, Qing Jiang

**Affiliations:** a State Key Laboratory of Pharmaceutical Biotechnology, Division of Sports Medicine and Adult Reconstructive Surgery, Department of Orthopedic Surgery, Drum Tower Hospital Affiliated to Medical School of Nanjing University, China; b School of Mechanical Engineering, Southeast University, China; c Hangzhou Lancet Robotics Company Ltd, China; d Taikang Xianlin Drum Tower Hospital, China; e Danyang Hospital of Traditional Chinese Medicine, China; f The Second People's Hospital of Xuanwei, China; g Cancer Hospital Chinese Academy of Medical Science, China; h The First Affiliated Hospital of Bengbu Medical College, China; i Xinxiang Central Hospital, China; j Lin Yi Hospital of Traditional Chinese Medicine, China; k Department of Radiology, Drum Tower Hospital Affiliated to Medical School of Nanjing University, China

**Keywords:** Meniscus injury, Deep learning model, MRI, Regional Convolutional Neural Network, AI, MRI, magnetic resonance imaging, FS FSE PDWI, fat-suppressed fast spin-echo proton density-weighted image, R-CNN, regional convolutional neural network, AI, artificial intelligence, PDW, proton density-weighted, CA, cartilage tissue, AH_tear, anterior horn tear, PH_tear, posterior horn tear, MBT, meniscus body tear, AD, anterior horn degeneration, PD, posterior horn degeneration, MBD, meniscus body degeneration, AH_intact, anterior horn health, PH_intact, posterior horn health, MBH, meniscus body health, RPN, region proposal network, ROI, region of interest, TP, true positive, FP, false positive, FN, false negative, AP, average precision, IoU, intersection over union

## Abstract

**Objective:**

Meniscus tear is a common problem in sports trauma, and its imaging diagnosis mainly relies on MRI. To improve the diagnostic accuracy and efficiency, a deep learning model was employed in this study and the identification efficiency was evaluated.

**Methods:**

Standard knee MRI images from 924 individual patients were used to complete the training, validation and testing processes. Mask regional convolutional neural network (R–CNN) was used to build the deep learning network structure, and ResNet50 was adopted to develop the backbone network. The deep learning model was trained and validated with a dataset containing 504 and 220 patients, respectively. Internal testing was performed based on a dataset of 200 patients, and 180 patients from 8 hospitals were regarded as an external dataset for model validation. Additionally, 40 patients who were diagnosed by the arthroscopic surgery were enrolled as the final test dataset.

**Results:**

After training and validation, the deep learning model effectively recognized healthy and injured menisci. Average precision for the three types of menisci (healthy, torn and degenerated menisci) ranged from 68% to 80%. Diagnostic accuracy for healthy, torn and degenerated menisci was 87.50%, 86.96%, and 84.78%, respectively. Validation results from external dataset demonstrated that the accuracy of diagnosing torn and intact meniscus tear through 3.0T MRI images was higher than 80%, while the accuracy verified by arthroscopic surgery was 87.50%.

**Conclusion:**

Mask R–CNN effectively identified and diagnosed meniscal injuries, especially for tears that occurred in different parts of the meniscus. The recognition ability was admirable, and the diagnostic accuracy could be further improved with increased training sample size. Therefore, this deep learning model showed great potential in diagnosing meniscus injuries.

**Translational potential of this article:**

Deep learning model exerted unique effect in terms of reducing doctors’ workload and improving diagnostic accuracy. Injured and healthy menisci could be more accurately identified and classified based on training and learning datasets. This model could also distinguish torn from degenerated menisci, making it an effective tool for MRI-assisted diagnosis of meniscus injuries in clinical practice.

## 1 Introduction

Meniscus is commonly referred to as the fibrocartilaginous structure located within the knee joint cavity, between the femur and tibia, providing strength to the joint and absorbing impact for protection [1,2]. It can be divided into medial meniscus and lateral meniscus. Meniscus injury is very common, with an incidence rate of 6–7 in 10,000 [3]. Destruction of meniscal integrity due to various conditions such as dysplasia, chronic strain, and acute sprains can lead to meniscal damage, accompanied by a series of clinical symptoms such as pain and dysfunction that severely impact the patient's mobility and quality of life. Once a meniscal injury is diagnosed, most of the cases need surgical treatment. Accurate and timely preoperative diagnosis is of great significance.

Magnetic resonance imaging (MRI) generates high imaging resolution of soft-tissue. This method allows a clear view of the shape and internal structure of the meniscus, and is the preferred examination for the diagnosis of meniscus injuries [4,5]. Fat-suppressed fast spin-echo proton density-weighted image (FS FSE PDWI), which produces homogeneous hypointense on MRI sequences, is most commonly used in the detection of meniscal injuries. A multi-center study showed that analyzing the risk and prognosis of meniscal injury had important clinical implications [6]. However, the accuracy of MRI diagnosis is limited due to the following reasons. Firstly, several irregularly shaped tissues are situated around the meniscus. Secondly, the abnormal signal of a meniscal tear is so small that it is not easy to be spotted on images. Thirdly, the amount of MRI data can be extremely huge (about 100 images per patient). Fourthly, the accuracy of diagnosis is influenced by the doctor's diagnostic experience. Furthermore, other subjective factors may also affect the diagnostic results.

In recent years, the application of artificial intelligence (AI) in the field of medical imaging has become a research hotspot, and it is believed that AI has the potential to provide accurate diagnosis and treatment. Deep learning and other AI applications can effectively improve the efficiency of data processing and reduce human errors through repetitive learning to identify disease patterns [7,8]. Traditional machine learning algorithms mainly include neural network, k-nearest neighbor, support vector machine, naive Bayes classifier, and random decision forest. These algorithms rely on the shallow features of artificial intelligence. One advantage of deep learning is that there is no need to specify the features manually, and machine can learn by itself through dataset training, bringing a breakthrough in image processing.

Great progress has been made in the in-depth analysis of knee MRI images using AI, but it is far less used in other critical conditions such as tumor, nerve damage and pulmonary nodules. Compared to bone and cartilage, the study on meniscus is limited because image segmentation and post-processing are not feasible. Among the AI studies regarding meniscal tears, most studies only analyzed the sagittal plane, and a few studies analyzed the sagittal plane, coronal plane, and cross-section simultaneously [9]. The areas under the curve (AUCs) for these studies ranged from 0.847 to 0.910 [10], meaning that this technology should be improved to increase the diagnostic accuracy by MRI.

Slice thickness is an important parameter in meniscus MRI examination. In previous studies, the scanning layer thickness ranged from 0.7 ​mm to 3.0 ​mm [5,11], which made the data sources lacking homogeneity. This study aimed to utilize the most commonly used sequences and scanning layer thicknesses in clinical practice for model training, providing a wider application range and benefiting future multi-center studies. After obtaining the feature map of meniscus MRI images through convolutional neural networks, Mask R–CNN was used to perform classification, regression, and pixel-level mask diagnosis. To verify the recognition accuracy of the deep learning model, the results were evaluated by experienced doctors in conjunction with arthroscopic surgery. We anticipated that this technology could serve as an effective tool for clinical MRI-assisted diagnosis of meniscal injuries.

## 2 Methods

### 2.1 Process of MRI scanning

This study followed relevant guidelines, and received approval from the ethics committee of Drum Tower Hospital affiliated to the Medical School of Nanjing University. All patients underwent MRI in a supine feet-first position using a 3.0 ​T ​MR imaging system (United Imaging Co., Ltd., Shanghai, China) with a dedicated knee coil. Sagittal fat-suppressed proton density-weighted (PDW) MR images were acquired digitally from the picture archiving and communication system (PACS; Neusoft Medical Systems Co., Ltd., Shenyang, China) in the Joint Photographic Experts Group (JPEG) format. The parameters of the MR FS PDW sequence were set as: 3 ​mm slice thickness, 0.3 ​mm gap, 1500 ​ms time of repetition, 40 ​ms time of echo, 16 ​× ​16 cm^2^ field of view, and 1 number of signal average. Sagittal position lines were set perpendicular to the line of the posterior femoral condyle on transverse images, and perpendicular to the articular surface of the tibial plateau on coronal images.

### 2.2 Inclusion criteria

According to the relevant clinical diagnostic guidelines, the menisci were divided into meniscus with tear and meniscus without tear. The diagnostic criteria for a meniscal tear included abnormal meniscal hyperintensity, and hyperintensity involving at least one articular surface of the meniscus or reaching the free edge of the meniscus [12]. Data sources of the study were obtained from the same MRI equipment and images were scanned by technicians with similar standardized training experience who were familiar with scanning parameters. In the image processing stage, the images without motion artifacts or any other magnetic artifacts were included.

### 2.3 Image dataset and masking

MRI image dataset was retrieved and produced by combining with clinical testing. For the recognition of fine results, the size of the acquired MRI images was selected as 1188 ​× ​1372 pixels. MRI images of 924 patients (18 images per patient) were collected and labeled to make the common objects in context datasets, in which 504 individuals were assigned into the training dataset group, 220 patients were in the verification dataset group, and 200 patients were in the internal testing dataset group. In addition, images of 180 patients from 8 hospitals were considered as an external testing dataset. To visualize the health of the menisci, the position and shape of the cartilage tissues (CA) were extracted and displayed from the images. The marking process was performed under the supervision of a board-certified radiologist and a board-certified sports medicine physician to ensure the accuracy of the marking range. Both of them had more than 15 years of experience in their respective fields.

**Fig. 1:**
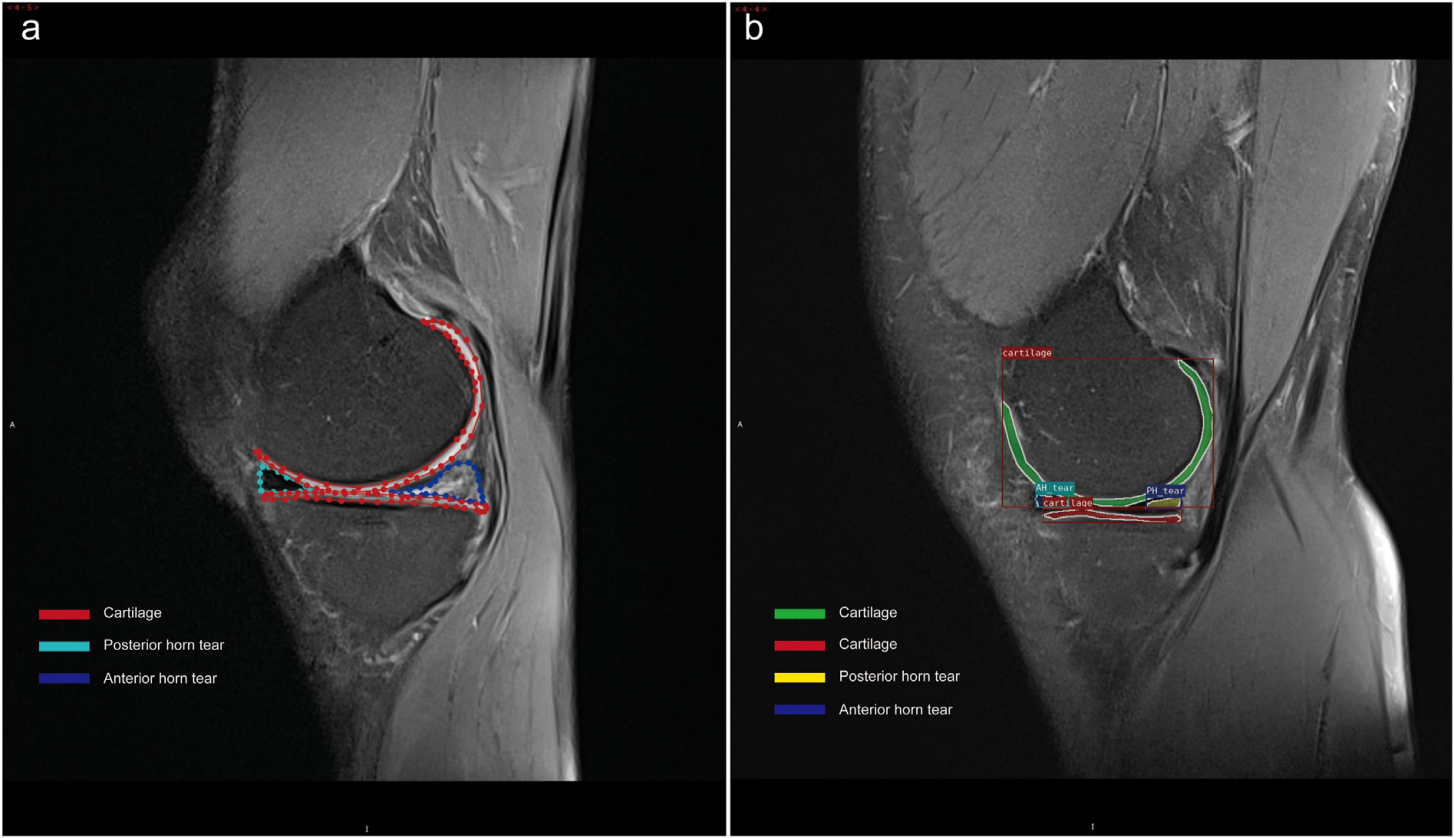
Meniscus MR Image dataset visualization process. (a) The marking process of objects on the image, (b) The exported image derived from the deep learning model.

MRI images were manually segmented into 10 categories: CA, anterior horn tear (AH_tear), posterior horn tear (PH_tear), meniscus body tear (MBT), anterior horn degeneration (AD), posterior horn degeneration (PD), meniscus body degeneration (MBD), anterior horn intact (AH_intact), posterior horn intact (PH_intact), and meniscus body health (MBH). During the marking and labeling process, cartilages without full display were discarded, and the pixels that could not be used to distinguish healthy from injured menisci were also ignored. Fig. 1 demonstrates the visualization process of the meniscus datasets. Usually, the cartilage was displayed clearly, and healthy and injured menisci were marked based on the doctor's diagnosis.

**Fig. 2:**
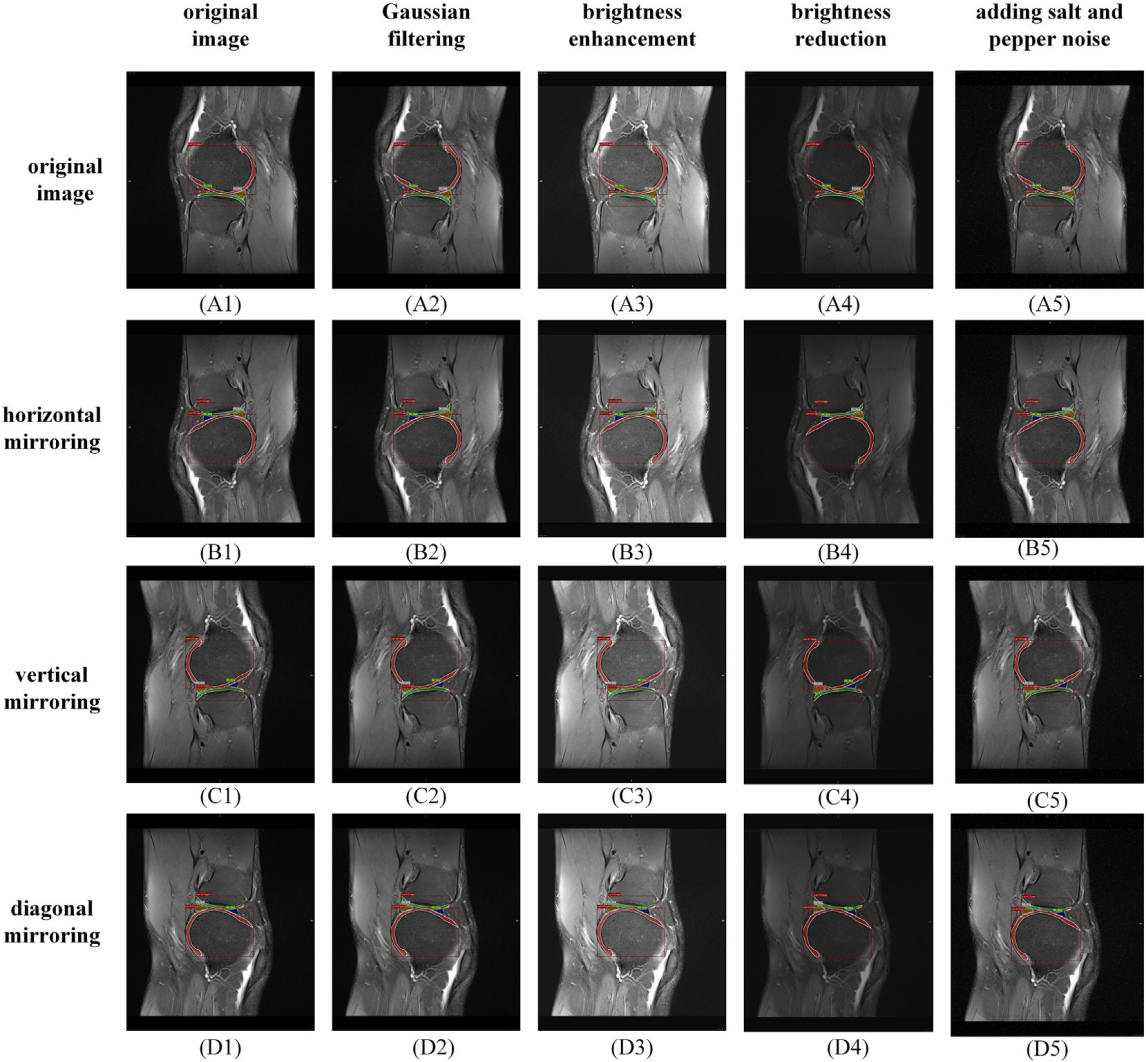
The illustration diagram of dataset augmentation technique.

Due to the limited number of patients, the amount of images in the dataset for training and validation might not be large enough. In the dataset establishment stage, data augmentation technology was used to supplement the collected dataset. Based on the labeled MRI image dataset, geometric transformation, lighting adjustment, Gaussian filtering and noise addition were used to expand the number of samples in the dataset. To prevent labeling errors, three geometric transformation methods were used, including horizontal, vertical and diagonal mirroring. By geometric transformation, one MRI image was converted into four different images. The four images were processed as follows: Gaussian filtering, brightness enhancement, brightness reduction and adding noise (such as salt and pepper noise). MRI images generated by geometric transformation could simulate differences due to various slice angles and positions. New images with different brightness could simulate different levels of fat suppression. Gaussian filtering blurred the original MRI images, and noise added increased the disturbance to images. As shown in Fig. 2, through data augmentation and labeling, the dataset was expanded by 20 times. Segmentation categories in the meniscus dataset are shown in Table 1. The total number of labels in the training, validation and internal testing dataset was 30080, 16520 and 1012, respectively.

**Table 1:** Meniscus dataset and demographic breakdown.

	Patients number	CA	PH_tear	AH_tear	MBT	PD	AD	MBD	AH_intact	PH_intact	MBH	Total
Training dataset	504	19780	1620	860	560	780	820	380	2660	2080	540	30080
Verification dataset	220	7260	1260	840	300	420	700	240	3020	1980	500	16520
Testing dataset	200	348	114	65	33	50	56	22	164	129	31	1012

### 2.4 Network architecture

**Fig. 3:**
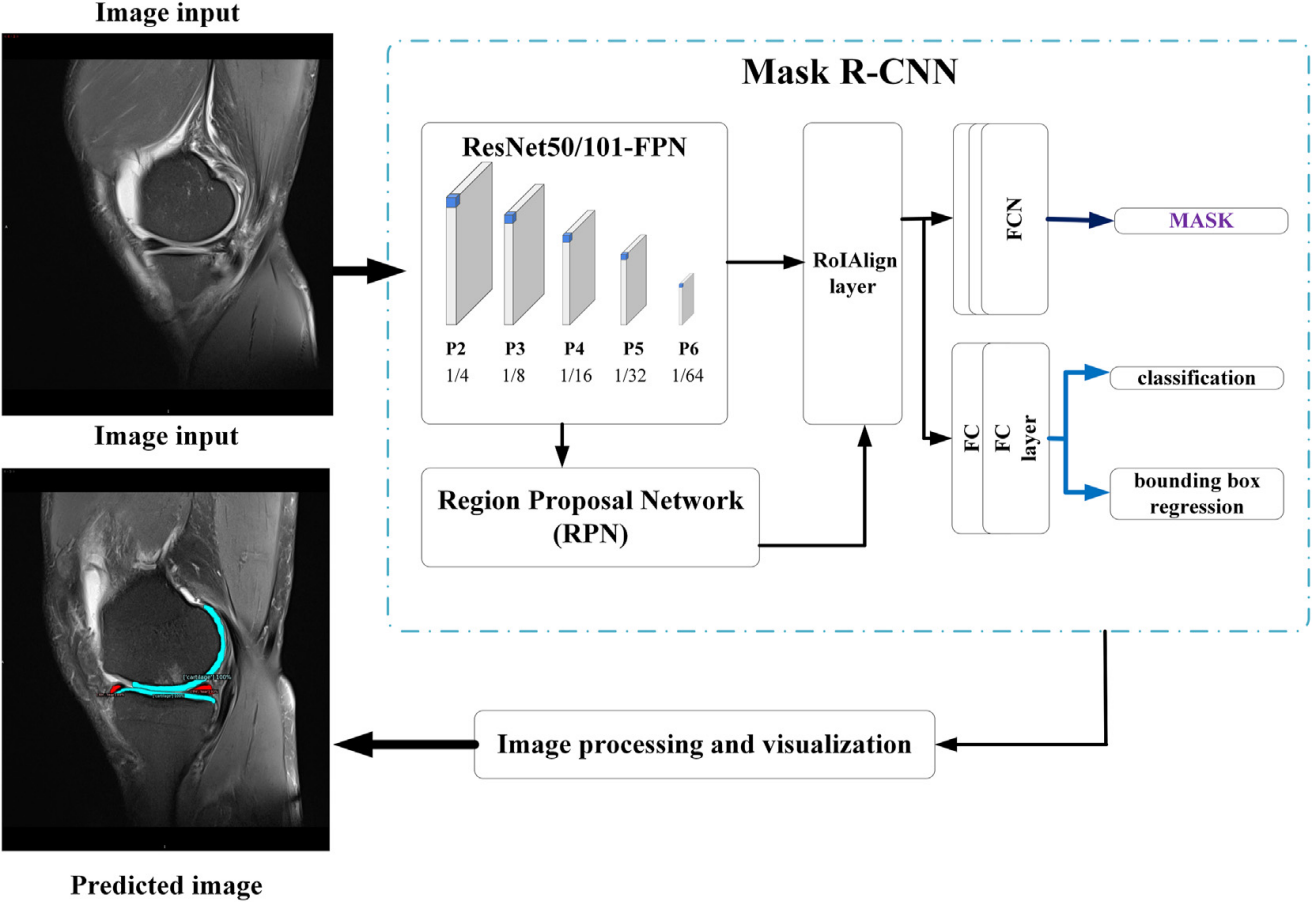
Architecture of the deep learning network for the identification of torn menisci.

In this process, Mask R–CNN was employed as the deep learning network structure to classify and segment the MRI images [13]. As shown in Fig. 3, the process of deep learning for the identification of meniscal injuries mainly included two stages. The first stage was the generation of candidate regions, which primarily included feature extraction by convolutional neural networks, Region Proposal Network (RPN) [14], and RoIAlign layer [13]. The second stage included object classification and regression and mask generation.

Feature map extraction was completed using ResNet50 architecture as the backbone network. Because ResNet50 had deeper network layers, it produced abundant feature information after convolution and pooling of the original images. ResNet50 was combined with Feature Pyramid Networks and feature maps from the bottom layer to the upper layer, which was conducive to making full use of the features of different depths [15]. The purpose of using RPN was to determine the region of interest (ROI) within the network. Briefly, the MRI image was input into the RPN, and the ROI of the original image was extracted by a 9-size anchor to output the region with a recommendation score. Bilinear interpolation was used in RoIAlign to extract fixed-sized feature maps (for example, 7 ​× ​7 pixel) from each ROI.

Mask R–CNN finally output three branches of the meniscus images: classification, bounding box regression, and a mask branch (Fully Convolutional Networks). In the dataset for the identification of meniscal injuries, the number of categories was 8 (including background and other 7 categories), the output depth of the classification and regression network was 8, and the output mask network size was 28 ​× ​28 ​× ​8 pixel.

### 2.5 Training

ResNet50 was adopted as the backbone network to train the MRI image datasets. Dataset was trained on a Graphic Processing Unit (RTX 2070; NVIDIA, Santa Clara, CA, USA) for 10000 epochs, the initial learning rate was 0.01 (drop with training), the IMS_PER_BATCH was 2, and the NUM_CLASSES was 8. During the training process, loss function was defined as:

(1) $$L=L_{rpn}+L_{mask}$$

where $\;L{\;}_{cls}$ and $L{\;}_{box}$ respectively represented the classification loss and bounding-box loss:

(2) $$\;L_{rpn}=L{\;}_{cls}\;+\;L{\;}_{box}=\frac{1}{N_{cls}}\underset{i}{\sum }L_{cls}\left(p_{i},p_{i}^{*}\right)+\;\lambda_{1}\frac{1}{N_{reg}}\underset{i}{\sum }p_{i}^{*}L_{reg}\left(t_{i},t_{i}^{*}\right)$$

where *N* represented the number of corresponding anchors or bounding boxes; the hyper-parameters λ and γ balanced the training losses of the regression and mask branch. $\;L{\;}_{cls}$ represented the classification loss function and was expressed as:

(3) $$L_{cls}\left(p_{i},p_{i}^{*}\right)=-\log\;p_{i}^{*}p_{i}$$

where *i* was the index of an anchor in a mini-batch; $p_{i}$ was the predicted classification probability of anchor *i*; $p_{i}^{*}$ represented the ground-truth label (correct and positive label) probability of the anchor *i*; $p_{i}^{*}$ was 1 for positive anchor and 0 for negative anchor.

$L{\;}_{box}$ was bounding-box loss defined over a tuple of true bounding-box regression targets:

(4) $$L_{reg}\left(t_{i},t_{i}^{*}\right)=smooth_{L1}\left(t_{i}^{*}-t_{i}\right)$$

(5) $$smooth_{L1}\left(x\right)=\left\{ \begin{array}{l} 0.5x^{2}\;,\;if\;\left|x\right|<1\\ \left|x\right|-0.5\;,\;otherwise \end{array}\right.$$

where $t_{i}^{*}=\left(t_{x}^{*},t_{y}^{*},t_{w}^{*},t_{h}^{*}\right)$ indicated the differences between the ground-truth label box and the positive anchor in four-parameter vectors (the horizontal and vertical coordinate values of the center point in the bounding box; the width and height of the bounding box); $t_{i}=\left(t_{x},t_{y},t_{w},t_{h}\right)$ represented the difference between the diagnostic bounding box and the ground-truth label box:

$$\;L{\;}_{mask}=L_{mask}\left(p_{i},p_{i}^{*},t_{i},t_{i}^{*},s_{i},s_{i}^{*}\right)$$

(6) $$=\frac{1}{N_{cls}}\underset{i}{\sum }L_{cls}\left(p_{i},p_{i}^{*}\right)+\lambda_{2}\frac{1}{N_{reg}}\underset{i}{\sum }p_{i}^{*}L_{reg}\left(t_{i},t_{i}^{*}\right)+\gamma_{2}\frac{1}{N_{mask}}\underset{i}{\sum }L_{mask}\left(s_{i},s_{i}^{*}\right)$$

Definition of $L{\;}_{mask}$ allowed the network to generate masks for every class without competition among classes. $L{\;}_{mask}$ was defined as the average binary cross-entropy loss used by a per-pixel sigmoid. Mask branch had a $\text{K}m^{2}$-dimensional output for each ROI (K was the number of classes). $\;L{\;}_{mask}$ was only defined on the k-th mask.

### 2.6 Model performance evaluation

To estimate the identification effect, MRI image testing dataset was used for testing and evaluation. Intersection over Union (IoU) was used, which was the ratio of intersection to union of candidate bound area (C) and ground truth bound area (G).

(7) $$\text{IoU}=\frac{area\left(C\right)\;\cap \;area\left(G\right)}{area\left(C\right)\;\cup \;area\left(G\right)}$$

Precision and recall of the formulas were:

(8) $$Precision=\frac{TP}{TP+FP}$$

(9) $$Recall=\frac{TP}{TP+FN}$$

where True Positive (TP) represented the resultant number of IoU values greater than the threshold values (generally 0.5). False Positive (FP) represented the number of IoU values less than the threshold values. False Negative (FN) represented the number of unrecognized targets.

Average Precision (AP) was used to measure the identification accuracy. For multi-class diagnosis, AP was the average precision of multiple categories. The formula was:

(10) $$AP=\overset{1}{\underset{0}{\int }}PdR\;\left(\text{IoU}=0.50\text{:}0.95\right)$$

AP50 and AP75 were APs when IoU threshold was greater than 0.5 and greater than 0.75, respectively. APs, APm, and APl were represented as the AP for small objects (area <32^2^), medium objects (32^2^<area <96^2^), and large objects (96^2^<area), respectively.

### 2.7 Diagnostic accuracy evaluation

The identification accuracy was evaluated by comparing the output results with the assessment by a board-certified radiologist with 15+ years of experience. Briefly, images from 200 patients having meniscus tears, meniscus degeneration, and intact meniscus were identified as the internal testing dataset using the AI model. Additionally, images from 180 patients with definitive MRI diagnosis reports from 8 hospitals were regarded as the external testing dataset. In accordance with routine diagnostic procedure, each MRI diagnostic report could be provided with the same diagnostic opinion by two board-certified radiologists to ensure its reliability. Among them, images from 90 patients (30 healthy, 30 with meniscus degeneration, and 30 with meniscus tear) were captured using 1.5T MRI, and 90 patients (30 healthy, 30 with meniscus degeneration, and 30 with meniscus tear) were scanned using 3.0T MRI. The scanning parameters are listed in Table 2. Different results between AI and manual reports were assessed by the experience radiologist. Moreover, 40 discharged patients who underwent arthroscopic surgery and MR test and were diagnosed with meniscus tear were randomly selected to verify the diagnostic accuracy of the AI model.

**Table 2:** MR imaging system and scanning parameters.

Types	Model	Field strength	MR sequence	Field of view	Time of repetition	Time of echo	Slice Thickness	Matrix
Philips	Intera	1.5	FS-T2W	18 ​cm∗18 ​cm	1800 ​ms	30 ​ms	4 ​mm	200∗160
United Imaging	uMR790	3.0	FS-PDW	16 ​cm∗16 ​cm	1500 ​ms	40 ​ms	3 ​mm	320∗288
Siemens	Skyra	3.0	FS-PDW	17 ​cm∗19.6 ​cm	2600 ​ms	36 ​ms	3.5 ​mm	384∗384
Siemens	Avanto	1.5	FS-PDW	16 ​cm∗16 ​cm	3000 ​ms	31 ​ms	4 ​mm	640∗640
Philips	Multiva	1.5	FS-PDW	16 ​cm∗16 ​cm	2000 ​ms	25 ​ms	4 ​mm	288∗224
GE	Architect	3.0	FS-PDW	16 ​cm∗16 ​cm	2500 ​ms	38 ​ms	4 ​mm	512∗512
Siemens	Avanto	1.5	FS-PDW	22.2 ​cm∗16.6 ​cm	2000 ​ms	19 ​ms	4.5 ​mm	640∗640
Siemens	Skyra	3.0	FS-PDW	16 ​cm∗16 ​cm	2800 ​ms	32 ​ms	3.5 ​mm	352∗288
GE	750	3.0	FS-PDW	18 ​cm∗18 ​cm	1941 ​ms	35 ​ms	3.5 ​mm	352∗224

## 3 Results

### 3.1 Mask R–CNN training

**Fig. 4:**
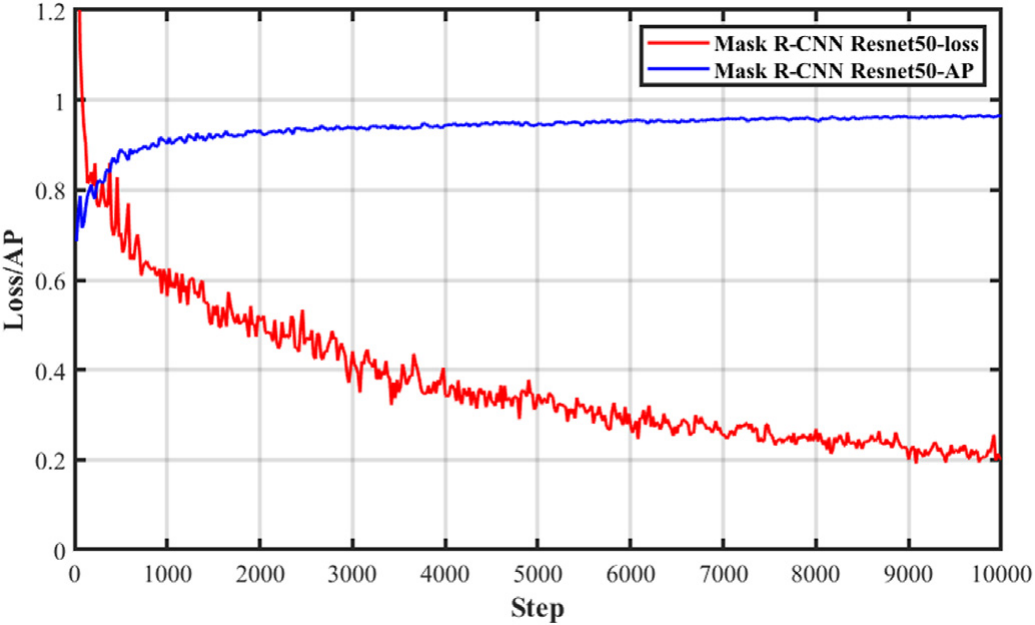
Loss function and accuracy in the training process of Mask R–CNN.

Loss function and accuracy in the training process of Mask R–CNN are shown in Fig. 4. After 10,000 iterations, loss function was relatively low, and the accuracy increased to 0.96. More training and larger datasets were conductive to improve accuracy and avoid overfitting.

### 3.2 Image identification of meniscus

**Fig. 5:**
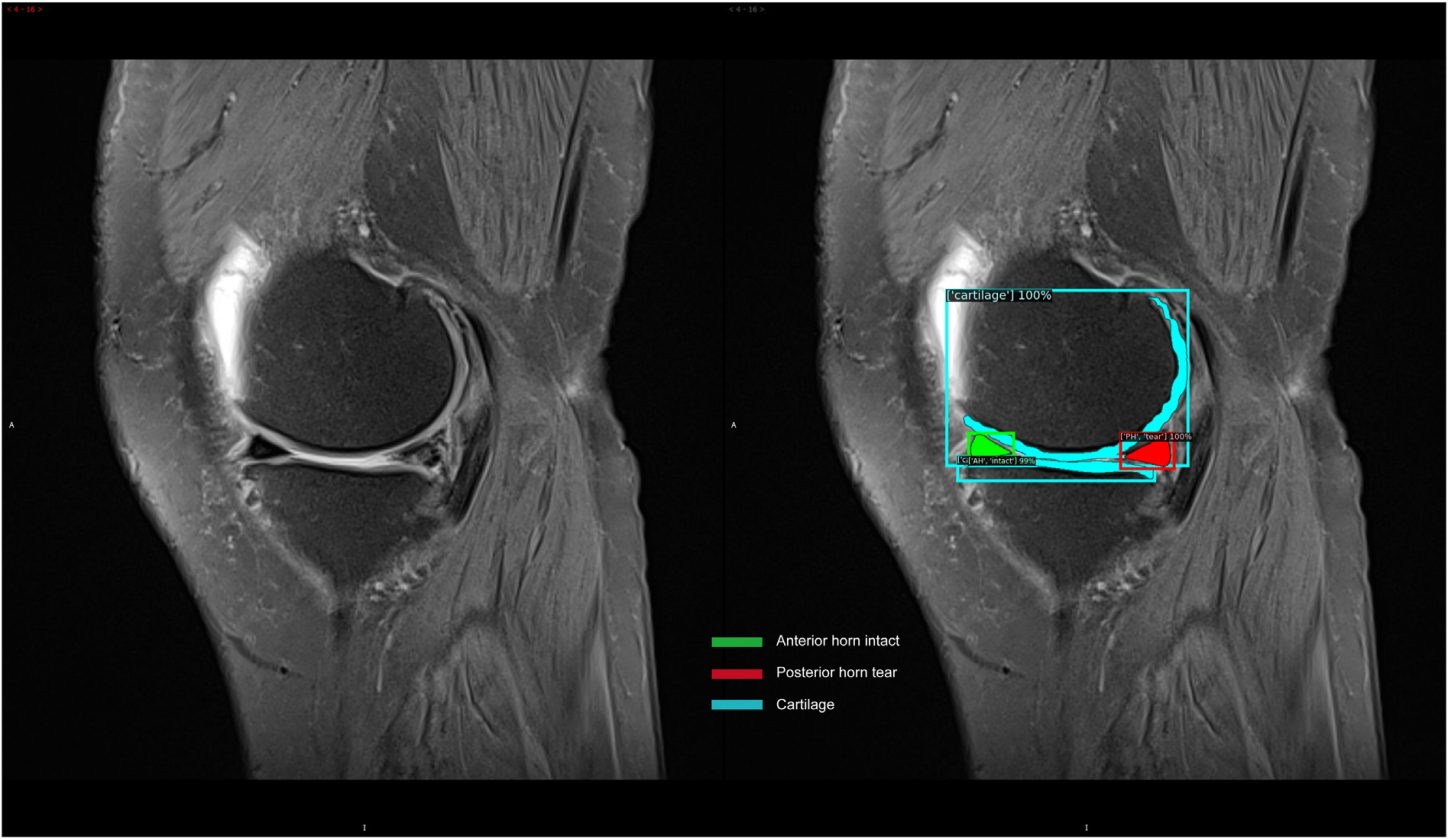
Classification and instance segmentation results of meniscus MR images.

**Fig. 6:**
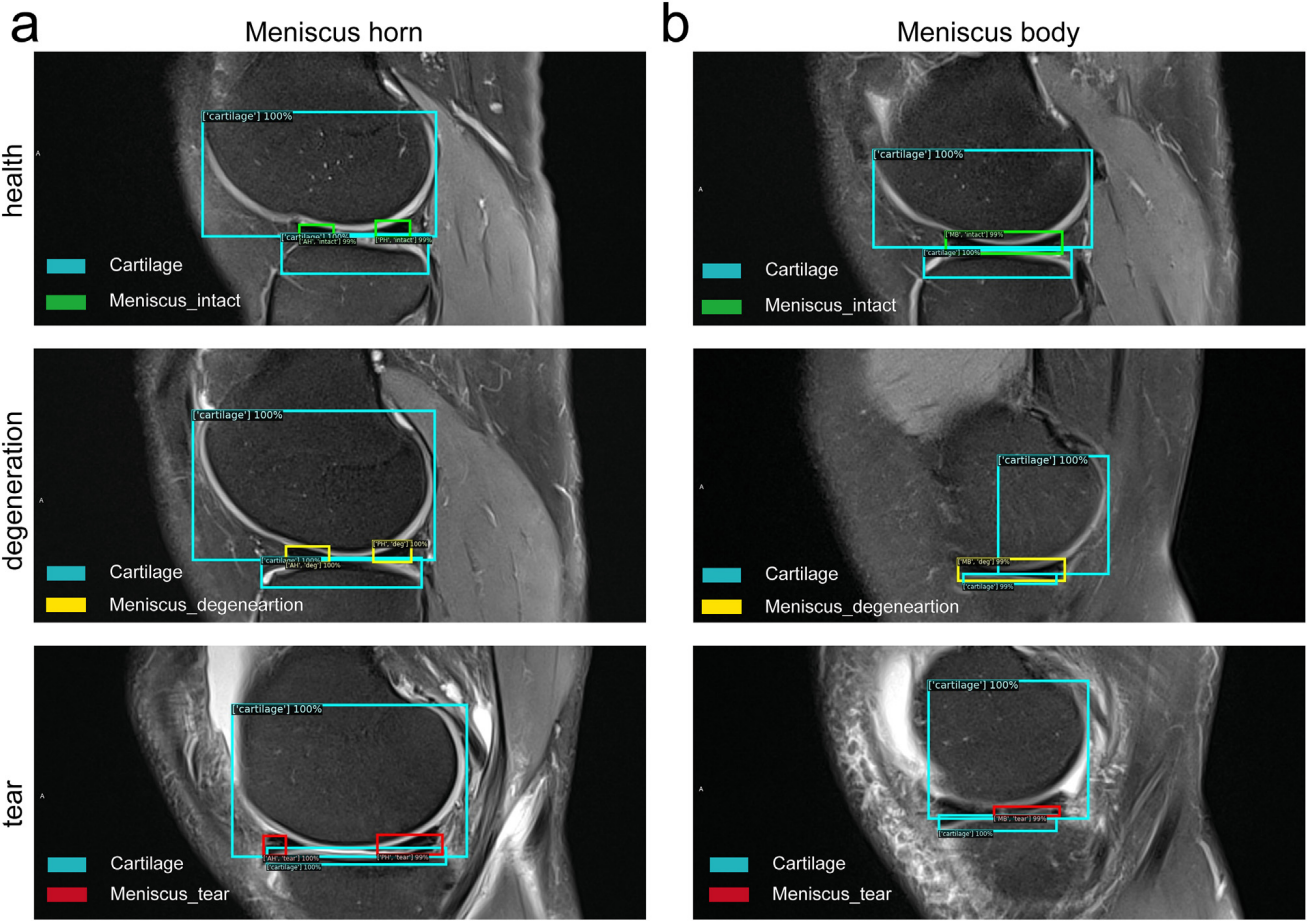
Bounding box diagnosis results on meniscus MR images. (a) Meniscal horns, (b) Meniscal body.

**Fig. 7:**
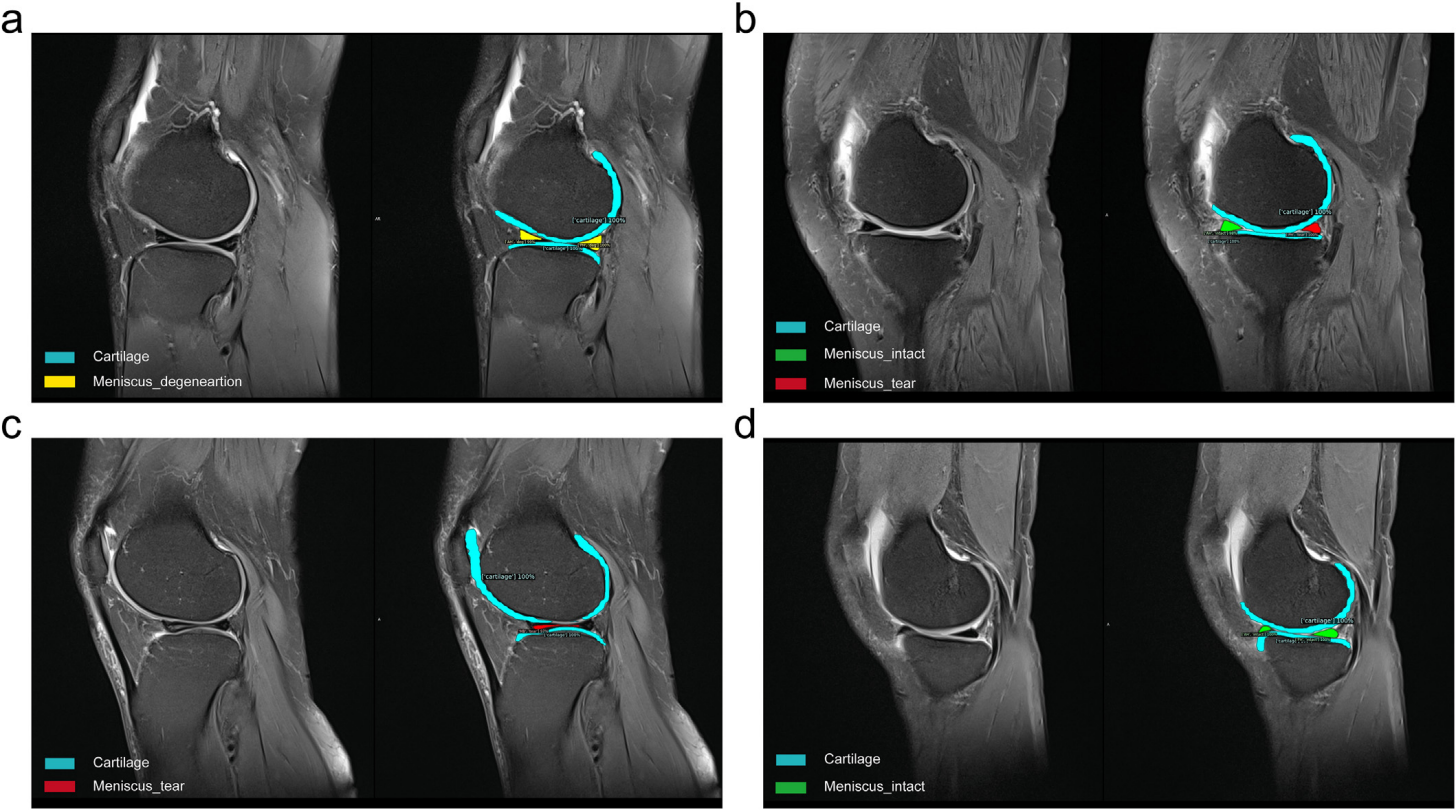
Mask diagnosis results on meniscus MR images. (a) Degenerations at meniscal anterior and posterior horns, (b) Tears at meniscal body, (c) Tears at the posterior horn, (d) Healthy meniscus.

Fig. 5 shows the classification and instance segmentation of a meniscus MR image, and the targeted objects were marked by both bounding box and pixel. The reorganization diagnosis results of Box and Mask are shown in Fig. 6 and Fig. 7, respectively. Box represented the bounding box containing the target objects (Fig. 6). Mask represented the predicted pixels of the tissues, like cartilage and meniscus (Fig. 7). Different colors were employed to distinguish the cartilage from meniscus: watchet blue bounding boxes and pixels represented CA, red bounding boxes and pixels represented injured menisci (PH_tear, AH_tear and MBT), yellow bounding boxes and pixels represented degenerated menisci (AD, MBD and PD), and green bounding boxes and pixels represented healthy menisci (AH_intact, MBH and PH_intact).

Box diagnosis results demonstrated that the meniscus horns (Fig. 6a) and the body (Fig. 6b) were precisely divided into three categories: healthy meniscus, degenerated meniscus, and meniscus with tear. Coverage of the CA on each layer was also included in the Box. According to Mask diagnosis results, degeneration occurred at both anterior and posterior horns of meniscus (Fig. 7a), tears occurred at posterior horns (Fig. 7b), tears occurred at the meniscus body (Fig. 7c), and healthy meniscus (Fig. 7d) was accurately identified.

AP of Mask R–CNN was tested with Resnet50_FPN as the backbone network, as exhibited in Table 3. The results indicated that when the IoU shoulder value was greater than 0.5, AP of Box and Mask was 99.55 ​± ​0.41% and 99.47 ​± ​0.28%, respectively. As the IoU threshold exceeded 0.75, Box and Mask APs dropped slightly but were still greater than 88%, exhibiting extremely high accuracy. APs for objects of different sizes were also acceptable, with all values above 50%. Due to the deep network layers of Resnet50, the AP identification accuracy was relatively good. For higher AP, the number of iterations was increased.

**Table 3:** AP for identification of meniscus injuries.

Backbone network	(%)	AP50	AP75	APs	APm	APl
Resnet50_FPN	Box	99.55 ​± ​0.41	97.67 ​± ​1.21	76.86 ​± ​4.82	82.07 ​± ​5.82	88.45 ​± ​4.11
	Mask	99.47 ​± ​0.28	88.15 ​± ​5.16	69.60 ​± ​5.33	74.99 ​± ​4.91	45.20 ​± ​6.56

### 3.3 Diagnosis of meniscus injures

Table 4 represents the AP evaluation results of each category in MRI images. Box AP of the CA was above 84%. Meniscus tear Box AP was higher than 68%, and the AP value for degenerated meniscus was greater than 79%. As for healthy meniscus, the AP value exceeded 80%. Although mask diagnosis was made at the pixel-level, the AP value of Mask was similar to that of Box.

**Table 4:** Per-category Box/Mask AP for identification of meniscus injuries.

Backbone network	(%)	CA	PT	AT	MBT	AD	MBD	PD	AH	MBH	PH
Resnet50_FPN	Box	84.64 ​± ​4.78	71.35 ​± ​3.66	68.84 ​± ​5.37	69.813 ​± ​3.49	82.84 ​± ​5.13	79.29 ​± ​4.46	84.56 ​± ​2.59	80.96 ​± ​6.23	82.58 ​± ​3.72	82.33 ​± ​3.98
	Mask	53.13 ​± ​6.39	75.50 ​± ​5.29	68.65 ​± ​4.72	63.69 ​± ​4.26	81.93 ​± ​3.92	75.91 ​± ​6.77	87.98 ​± ​5.13	80.22 ​± ​4.81	74.31 ​± ​4.46	83.38 ​± ​4.16

Sensitivity results at IoU ranging from 0.50 to 0.95 are shown in Table 5. Overall sensitivity for Box and Mask was 83.77 ​± ​5.29% and 74.43 ​± ​3.41%, respectively. Sensitivity for target objects in different areas was also admirable. For small- and medium-sized areas, the values were all above 75%. For large areas, the sensitivity for Box was as high as 95.77 ​± ​2.89%. Since the detected objects were relatively concentrated in small and medium areas, Mask sensitivity for large area was relatively low, but still exceeded the critical value of 50%.

**Table 5:** Sensitivity for identification of meniscus injuries.

Backbone Network	(%)	Overall Sensitivity	Area ​= ​Small	Area ​= ​Medium	Area ​= ​Large
Resnet50_FPN	Box	83.77 ​± ​5.29	78.16 ​± ​3.37	86.30 ​± ​5.28	95.77 ​± ​2.89
	Mask	74.43 ​± ​3.41	73.54 ​± ​4.92	78.22 ​± ​4.36	59.67 ​± ​2.72

Compared to the diagnosis by experienced doctors, the identification and diagnosis accuracy of the model was also quite high. Among the 200 patients from the internal testing dataset, 6 samples were unrecognized (3%). Besides, 49 of 56 healthy samples (including 3 unrecognized torn and 4 unrecognized degenerated samples), 80 of 92 torn samples (including 3 unrecognized healthy and 9 unrecognized degenerated samples), and 39 of 46 degenerated samples (including 3 unrecognized torn and 2 unrecognized healthy samples) were identified by deep learning model. Therefore, the diagnostic accuracy was 87.50% for healthy meniscus, 86.96% for torn meniscus, and 84.78% for degenerated meniscus.

For external testing dataset, the 3.0T MRI group demonstrated better recognition rate and diagnostic accuracy compared to the 1.5T MRI group (Table 6). Briefly, the healthy meniscus showed the highest recognition rate in both the 3.0T and the 1.5T MRI groups. The lowest recognition rate was found for degenerated meniscus by using MRI with both field strength. Torn meniscus could be effectively diagnosed in the 3.0T MRI group, but 1.5T MRI revealed the lowest diagnostic accuracy for torn meniscus among the three meniscus types.

**Table 6:** Verification of external dataset.

Field strength	Meniscus type	Recognition rate	diagnostic accuracy
3.0	Intact	93.33% (28 of 30)	82.14% (23 of 28)
	Degeneration	76.67% (23 of 30)	73.91% (17 of 23)
	Tear	86.67% (26 of 30)	92.31% (24 of 26)
1.5	Intact	80.00% (24 of 30)	79.17% (19 of 24)
	Degeneration	66.67% (20 of 30)	70.00% (14 of 20)
	Tear	76.67% (23 of 30)	60.87% (14 of 23)

The verification results of arthroscopic surgery were also optimistic, among 40 patients with a diagnosis confirmed by the gold standard, 87.50% of them (35 of 40) obtained corrected diagnosis using this model.

## 4 Discussion

Meniscal injury is one of the most common sports injuries worldwide [16]. MRI test generates a high soft-tissue image resolution, and is the first diagnostic test choice for meniscal injury [3]. However, the diagnostic accuracy of meniscal injury depends on the experience of the diagnostician. The popularization of multi-center studies on meniscus injury is hampered by the objective criteria of diagnosis, the subjective errors of doctors, and the diagnostic efficiency. These limitations put forward the objective demands on standardized interpretation and automated classification of meniscal MRI images. Herein, we proposed a deep learning network based on Mask R–CNN to address the demands mentioned above. We adopted the most commonly used conventional sequence and routine scanning parameters to ensure that the model could be widely used in different hospitals, and the anatomical images generated from different MR machines were consistent. The results proved that this method could recognize the injured meniscus with high accuracy. Thus, we believed that this deep learning network could be regarded as an effective measure in clinical application.

We used a deep learning method based on Mask R–CNN to realize identification and diagnosis of meniscal injuries. After annotation and classification, MRI images from 924 patients were collected. Substantial amount of images (nearly twenty thousands) were used in this study with admirable sensitivity and diagnostic accuracy. The images were segmented into 10 categories to estimate meniscus injuries. Compared to similar studies using deep learning model to diagnose meniscus injuries, this study had the largest label numbers (Table 7), indicating that the model could help the doctors to recognize more subjects on MRI images [[17], [18], [19], [20], [21], [22]]. In addition, although most of the previous studies could reach a sensitivity of higher than 90%, the verifying process was performed using the internal dataset. Only one study enrolled an external dataset to verify the effect of the model, and the sensitivity was 81% [22], which was slightly lower than that in our study. Besides, only one or a few images were selected from each patient for the training dataset in some studies [18,19,22]. These images showed typical features of healthy or torn meniscus, which might lead to missed diagnosis of meniscal injuries with non-typical characteristics or not appearing in the corresponding scan plane. Moreover, these studies did not take into account the distinction between degenerated and torn meniscus, which was difficult to diagnose clinically, even by experienced radiologists. The model established in this study could assist the radiologists to improve the diagnostic accuracy for meniscus degeneration and meniscus tear.

**Table 7:** Comparison of AI studies for meniscus tear diagnosis.

Study	Reference standard	Label No.	Network structure	Sequences	Field strength	Patients No.	Image No.	Verification method
This study	Radiologists/Arthroscopic surgery	10 (intact/tear/degeneration/horn/body/cartilage//anterior/posterior)	ResNet	SAG FS PDW, SAG FS T2	1.5/3.0	1104	19872	External dataset (87.50%)
Bien et al. [17]	Radiologists	2 (intact/tear)	MRNet	SAG T2, COR T1, ax PD	1.5/3.0	1088	≈33000	Internal dataset (74.10%)
Couteaux et al. [18]	Radiologists	4 (intact/tear/anterior/posterior)	ConvNet	FS-T2W	3.0	/	1128	Internal dataset (90.60%)
Roblot et al. [19]	Radiologists	3 (intact/horizontal tear/vertical tear)	Fast-RCNN/faster-RCNN	SAG T2	1.5/3.0	/	1123	Internal dataset (90.00%)
Pedoia et al. [20]	Radiologists	2 (intact/tear)	U-Net	SAG 3D PDW COR and SAG FS sensitive MRI	3.0	302	1478	Internal dataset (89.81%)
Fritz et al. [21]	Arthroscopic surgery	2 (intact/tear)	DCNN	COR and SAG FS fluid-sensitive MRI	1.5/3.0	100	20520	Internal dataset (91.20%)
Rizk et al. [22]	Radiologists	2 (intact/tear)	MRNet	SAG FS PDW, COR FS PD	1.0/1.5/3.0	10401	11353	External dataset (81.00%)

In the deep learning networks, Resnet50_FPN was used as backbone network, which had more network layers to integrate the features of the image at different depths. RPN used a 9-size anchor to extract the ROI from the original image, and output category scores and box scores. Mask R–CNN finally delivered classification, bounding box regression and mask diagnosis. After realizing classification and recognition, the pixel-level diagnosis with MRI was carried out. Through the training on MRI image datasets, AP of the bounding box regression was greater than 97%, and AP of pixel-level diagnosis was greater than 88%. In the visualized diagnosis results, the size and location of the meniscus injury were displayed and marked, which facilitated better diagnostic estimation.

In the validating process, three types of menisci were well identified. The most common problem was mistaking a degenerated meniscus as a torn meniscus. According to the imaging principle, there might be two main reasons for this misidentification. The first reason was similar signal intensity. Meniscal degeneration and meniscal cleft were actually at different stages of the same pathological process. The distinction between the two stages was not obvious, leading to the confusion between severe meniscal degeneration and meniscal spallation [23,24]. The second reason was related to the complex anatomy of meniscus. The shape of meniscus was irregular, its free edge was very slender with a thickness less than 0.5 ​mm, which was not easy to identify. In addition, when a meniscal tear occurred, the broken fragments could shift to the femoral intercondylar notch, and the original anatomical area of the meniscus was replaced by fluid signal, which might result in erroneous recognition.

Except the validation using internal dataset, images of 180 samples from 8 hospitals scanned by MRI with different brands and field strength were used as the external dataset. The results demonstrated that this model had admirable recognition rate in 3.0T group, especially for intact and torn menisci. But the recognition and diagnosis accuracy of the 1.5T group was not as good as those of the 3.0T group. One possible reason was that the images obtained by the 1.5T MRI equipment were not employed in the learning and training process. The image quality parameters including matrix and signal noise ratio differed between the 1.5T and 3.0T images. The recognition rate for degenerated meniscus was limited due to the ambiguous boundary between degeneration and tear. In addition, images of 40 samples diagnosed as meniscus tear through arthroscopic surgery were tested by this model to further verify the reliability of the results, and the diagnostic accuracy rate was nearly 90%. Of the relevant studies, only one of them employed arthroscopic surgery as the reference standard. The introducing of this gold standard could improve the confidence of orthopaedic specialists in applying this model and promote the clinical application of this technique.

Artificial intelligence has a broad application prospect for efficient analysis and classification of medical images. At present, there are great challenges in the application of artificial intelligence in diagnosing knee joint abnormalities. Except for the model algorithm and other technical factors, the biggest challenge is to establish a homogenized standard dataset, and adopt it on different people and by different MRI equipment. This requires a trade-off between diagnostic accuracy and generalizability. Herein, in this study, the training dataset was only collected from one 3.0T MRI equipment using the general scanning sequence. To enhance the learning and training effects, data augmentation technique was used in the training process. This technique could simulate different slice angle, position, and fat suppression level by using geometric transformation, lighting adjustment, Gaussian filtering and noise addition. Through this operation, the dataset could be expanded by 20 times, implying that the images number reached hundreds of thousands. But more importantly, the dataset could be further expanded by modifying the parameters of the currently used data augmentation methods.

**Figure 8:**
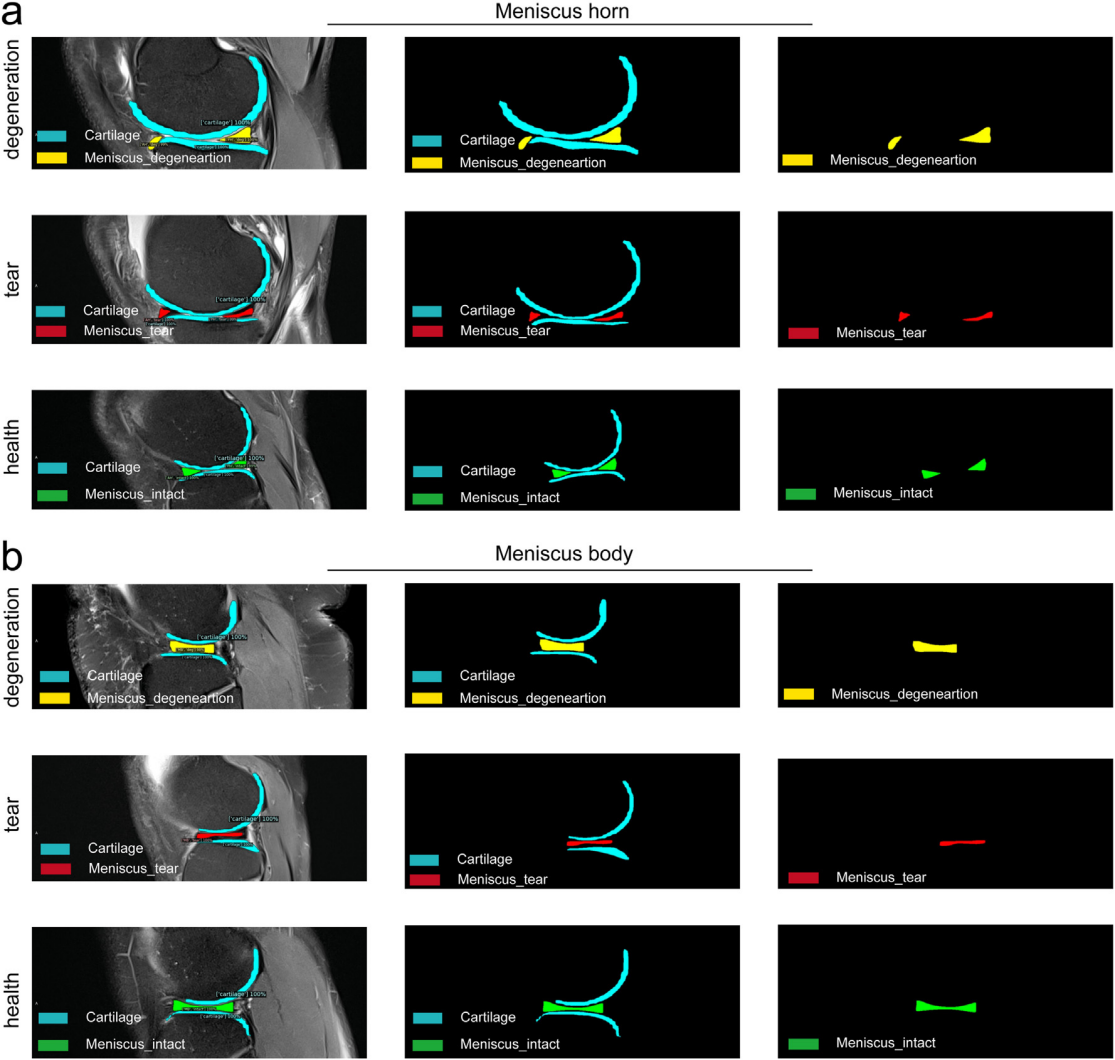
Diagnostic result highlighting and processing on meniscus MR images. (a) Meniscal horns, (b) Meniscal body.

In addition to improving diagnostic accuracy, AI can also help doctors clearly distinguish the diagnosis. Herein, image processing was performed to highlight the health status of meniscus. As shown in Fig. 8, after removing the soft tissue background and cartilage, the deep learning model could differentiate the situation of the meniscus. The green, yellow, and red pixels respectively represented healthy, degenerative, and torn meniscus. The horns (Fig. 8a) and body (Fig. 8b) of the meniscus, and the cartilage were easily recognized in this process.

There were several limitations in this study. First, only meniscal injury was identified, and no distinction was made between different types of meniscus tears. Moreover, the diagnostic accuracy of sagittal, coronal, and transverse views was not compared in the analysis. In future studies, we will analyze the accuracy of this model for meniscus tears at different positions by including more cases and investigating the influence of different layer thicknesses on the diagnosis of meniscus tears using AI. Additionally, the validation method also needed to be improved. Only a few sample diagnoses in this study were confirmed by arthroscopic surgery. This result might partially reflect the effect of the deep learning model, but was not statistically representative. In future research, rigorous comparison of arthroscopic diagnostic results with the data used for training and verification is required to further improve the accuracy of the model.

## 5 Conclusion

In summary, a Mask R–CNN model was employed in this study to identify and predict meniscus tears on MRI images. This deep learning model effectively detected the meniscus and cartilage, especially tears that occurred at different parts of the meniscus. The recognition accuracy was greater than 84%. With increased training sample size, the diagnostic accuracy could be further improved. The application of this technique may help reduce the misdiagnosis rate of meniscus injuries and alleviate the burden on doctors.

## Declaration of competing interest

The authors have no conflict of interest.
